# Recent Developments of Magnetoresistive Sensors for Industrial Applications

**DOI:** 10.3390/s151128665

**Published:** 2015-11-12

**Authors:** Lisa Jogschies, Daniel Klaas, Rahel Kruppe, Johannes Rittinger, Piriya Taptimthong, Anja Wienecke, Lutz Rissing, Marc Christopher Wurz

**Affiliations:** Institute of Micro Production Technology, Centre for Production Technology, Leibniz Universitaet Hannover, Garbsen 30823, Germany; E-Mails: jogschies@impt.uni-hannover.de (L.J.); klaas@impt.uni-hannover.de (D.K.); kruppe@impt.uni-hannover.de (R.K.); rittinger@impt.uni-hannover.de (J.R.); taptimthong@impt.uni-hannover.de (P.T.); anja.wienecke@gmx.net (A.W.); rissing@impt.uni-hannover.de (L.R.)

**Keywords:** magnetoresistive sensors, AMR, GMR, new magnetic sensor concepts, magnetic sensors for flexible electronics

## Abstract

The research and development in the field of magnetoresistive sensors has played an important role in the last few decades. Here, the authors give an introduction to the fundamentals of the anisotropic magnetoresistive (AMR) and the giant magnetoresistive (GMR) effect as well as an overview of various types of sensors in industrial applications. In addition, the authors present their recent work in this field, ranging from sensor systems fabricated on traditional substrate materials like silicon (Si), over new fabrication techniques for magnetoresistive sensors on flexible substrates for special applications, e.g., a flexible write head for component integrated data storage, micro-stamping of sensors on arbitrary surfaces or three dimensional sensing under extreme conditions (restricted mounting space in motor air gap, high temperatures during geothermal drilling).

## 1. Introduction

The anisotropic magnetoresistive (AMR) effect was first described in 1857 by William Thomson [1]. Thomson observed that the resistivity of ferromagnetic materials depends on the angle between the direction of electric current and the orientation of magnetization. First technical applications of this effect were introduced 100 years later, when the theoretical and practical prerequisites for the realization of thin film AMR sensors were obtained. A brief depiction of the essential basics of the AMR effect in thin films will be given in Section 2. 

In the following years, AMR sensors were primarily used as read heads in magnetic hard disk drives. Thanks to their simplicity of design, low cost, robustness and temperature stability, they were introduced in a wide range of industrial applications, including automotive, consumer electronics or biotechnology. In automotive and consumer electronic applications, magnetoresistive (MR) sensors are used for current sensing or position, speed and angle sensing as well as Earth’s magnetic field sensing in compass applications. In biotechnology, MR sensors are used for bimolecular detection in protein assays using magnetic tags or in microfluidic systems for magnetic bead manipulation [2,3,4,5]. An overview on designs and commercial devices of AMR sensors are given in Section 3. Another magnetoresistive effect was discovered in 1988 in thin ferromagnetic films [6]. This effect was denominated giant magnetoresistive effect (GMR), because the measured change of magnetoresistance largely exceled that of the AMR effect. The GMR effect occurs in a multilayer sandwich of two magnetic layers that are separated by a thin non-magnetic film. The large change of magnetoresistance is explained in literature as a scattering of electrons when they pass through the non-magnetic interface. More details on the underlying mechanisms of the GMR effect are given in Section 4. Examples of commercial devices of GMR sensors are depicted in Section 5.

In addition, we present current research in the field of MR-based sensors for specific industrial applications in Section 6. We conclude with an outlook on the perspectives and new fields of application for MR based sensors.

## 2. The Anisotropic Magnetoresistive (AMR) Effect in Thin Films

The AMR effect occurs in *3d* transition metals and can be observed macroscopically by a change of resistivity when a magnetic field is applied on a current-carrying sample of such material. This directional dependence of the magnetic properties of such material is denominated magnetic anisotropy. 

On the atomic level, the occurrence of the AMR effect can be explained as a consequence of the specific band structure in ferromagnetic metals. In these materials, the *3d* band is not fully filled and *4s* electrons are likely to be scattered to the *3d* sub-bands when a magnetic field is applied. The anisotropy of the magnetoresistance can be explained by the asymmetry of the electron orbits, which leads to differing scattering cross-sections of conducting electrons travelling either parallel or perpendicular to the direction of magnetization. The asymmetry of electron orbits is in turn a consequence of spin-orbit coupling. The fundamentals of these electric transport characteristics have been studied in detail in many papers since the 1960s, a comprehensive summary can be found in [7]. 

On the microscopic level, different sources that cause magnetic anisotropy can be distinguished [8]:

- Magnetocrystalline anisotropy: Directional dependence of magnetic properties due to the crystalline structure of the sample.
- Shape anisotropy: Directional dependence of magnetic properties due to the outer shape of the sample.
- Magnetoelastic anisotropy: Tensions cause a change of the magnetic behavior of the sample.
- Exchange anisotropy: A result of interactions between antiferromagnetic and ferromagnetic materials. Does not occur in AMR sensors, since no antiferromagnetic materials are used.

Due to their band structure, all ferromagnetic materials exhibit strong internal magnetization. However, this magnetization is only homogeneously directed in small, limited volumes, the so-called magnetic domains. In a polycrystalline bulk material, the magnetization of these domains is randomly distributed in all spatial directions. In a ferromagnetic thin film, the thickness of the film is small in comparison to the planar extent of the domains. 

**Figure 1:**
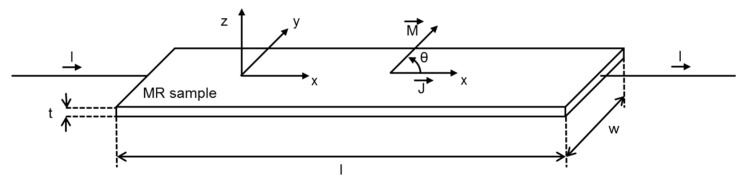
Schematic of an AMR thin film element.

Due to this shape anisotropy, the demagnetization factor perpendicular to the film plane is strongly elevated and the internal magnetization can be considered to be oriented in the film plane. This is the case for AMR based sensors; the change of magnetoresistance can thus be treated as a two-dimensional problem. Considering the coordinate system and MR sample depicted in Figure 1, the tensor of resistivity can be simplified to

(1) $$\text{ρ}=\left[\begin{array}{cc} {\text{ρ}}_{\|} & 0\\ 0 & {\text{ρ}}_{⊥} \end{array}\right]$$

As a consequence, the change of resistivity of a thin film sample solely depends on the angle θ between the direction of electric current $\vec{J}$ and the orientation of its internal magnetization $\vec{M}$ [7]. In the simplified case of a single domain, thin film element with a distinctive, well-defined orientation of magnetization, the change of resistivity ρ can be described by the following formula, where $\text{ρ}={\text{ρ}}_{\|}$ for θ=0° and $\text{ρ}={\text{ρ}}_{⊥}$ for θ=90°:

(2) $$\text{ρ}\left(\text{θ}\right)={\text{ρ}}_{⊥}+\left({\text{ρ}}_{\|}-{\text{ρ}}_{⊥}\right)\cos^{2}\text{θ}={\text{ρ}}_{⊥}+\Delta \text{ρ }\cos^{2}\text{θ}$$

The ratio $\Delta \text{ρ}/{\text{ρ}}_{\|}$ is called the magnetoresistive coefficient and a central term to evaluate the performance of a magnetoresistive sensor device. At room temperature, the magnetoresistive coefficient amounts to a range of a few percent for NiFe alloys [9]. A widely used material is the alloy NiFe 81/19 due to its magnetostriction constants close to zero in all crystal directions. 

## 3. Designs and Commercial Solutions of AMR Sensors

**Figure 2:**
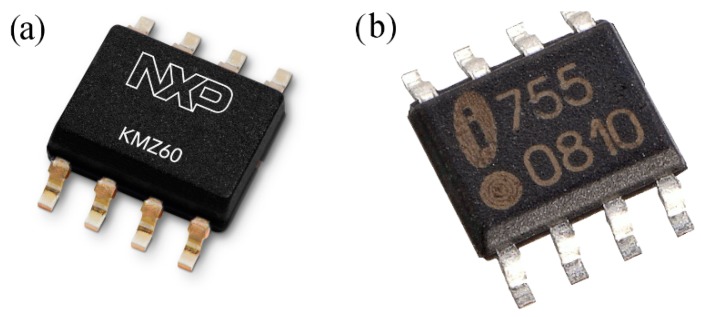
Examples of commercial AMR sensor devices: (**a**) NXP KMZ 60 angle sensor [10]; and (**b**) Sensitec GmbH AFF755B field sensor [11].

Due to the correlation described by Equation (2), thin film elements of ferromagnetic materials can be used as contactless angle or rotation sensors. Sensors for angle and rotation measurements are designed for rather strong magnetic fields, in order to lower the impact of interfering magnetic fields. Nonetheless, AMR sensors can also be used for the measurement of rather low magnetic fields like the Earth’s magnetic field, permitting the use of AMR sensors as compasses. A number of suppliers offer a large variety of commercially available devices (see Figure 2) [10,11].

In these devices, the thin film elements are usually operated in a Wheatstone bridge in order to compensate temperature drift and to double the signal output. 

The single thin film elements typically feature meander shaped geometry for two main reasons: First of all, this shape induces a strong magnetic anisotropy, providing the sensor with a well-defined orientation of sensitivity. Second, the length of the sensing element is increased, thus the absolute value of the change of resistance rises as well. This improves the sensitivity of the sensor. The crystal structure of the thin film is optimized in order to achieve a strong anisotropy within the film plane as well. The crystallographic orientation is determined by the deposition process as well as the parameters used for the deposition. Furthermore, it can be influenced by the application of a thin underlayer such as chromium [12]. 

An aspect often ignored is the impact of magnetoelastic anisotropy on the performance of AMR sensors. This is acceptable for commercial devices, when the ferromagnetic thin films are applied on rigid and very smooth surfaces, such as silicon or silicon oxide. However, when the thin films are applied onto rather rough and flexible substrates, the impact of the magnetoelastic anisotropy is no longer negligible. This issue will be addressed in detail in Section 6. 

**Figure 3:**
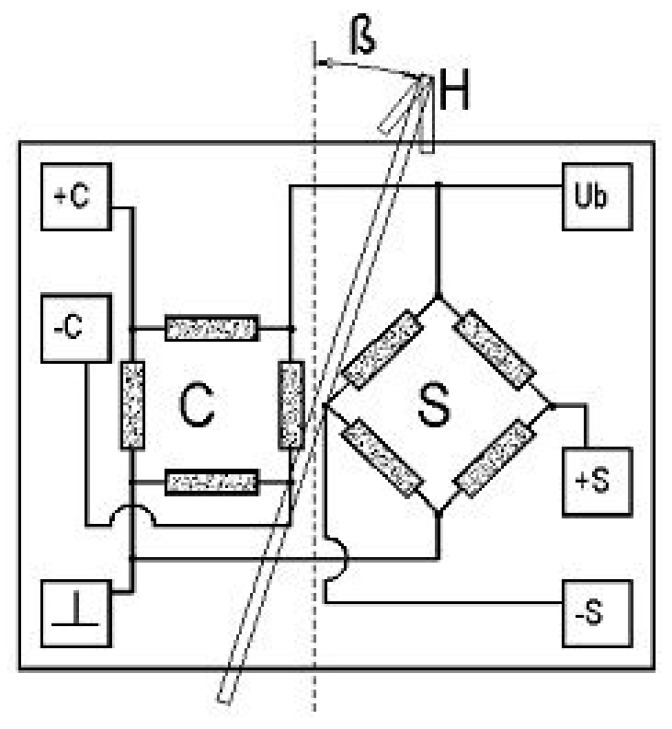
Schematic of a typical structure of a rotational AMR sensor [13].

The signal output of a single Wheatstone bridge of AMR elements correlates as well to a quadratic trigonometric function. Thus, only an angle of 180° can be monitored unambiguously. For the observation of a rotation of 360°, commercial devices for rotational speed or angle sensing are composed of two Wheatstone bridges that are shifted by 45° to each other (see Figure 3).

## 4. The Giant Magnetoresistive (GMR) Effect in Thin Films and Granular Alloys

**Figure 4:**
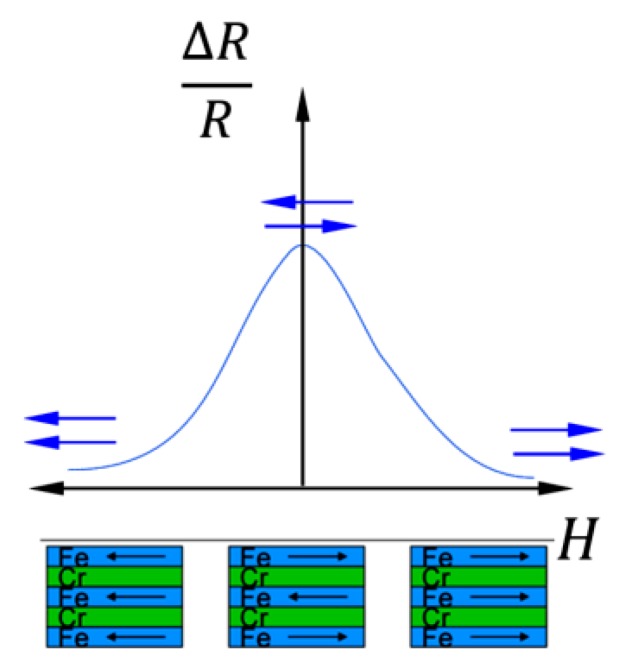
Antiparallel and parallel aligned layer stacks with related resistance dependence (after [15]).

The giant magnetoresistive (GMR) effect was discovered in 1988 in multilayered structures of ferromagnetic (fm) and non-ferromagnetic (nfm) thin films by Grünberg, Binasch *et al.* [14] as well as Fert, Baibich *et al*. [15]. They observed a change in the electrical resistance of a multilayer stack where the fm layers were coupled via interlayer exchange coupling (IEC) through a metallic nonmagnetic spacer layer (oscillatory dependence of relative magnetization orientation on the spacer layer thickness, discovered only a few years earlier in 1986 [16], although the oscillatory character was first experimentally proven 1990 by Parkin *et al*. [17]) dependent on the relative orientation of magnetization in the ferromagnetic layers. When aligning the magnetization directions of the fm layers from the initial antiparallel state to a parallel configuration by applying an external magnetic field, the electrical resistance of the layer stack decreased (Figure 4). 

The phenomenological description of this effect is given by

(3) $$\text{𝐺MR = }\frac{{\text{ρ}}_{ap}-{\text{ρ}}_{p}}{{\text{ρ}}_{p}}=\;\frac{{\text{σ}}_{p}}{{\text{σ}}_{ap}}-1$$

where ρ is the resistivity and σ the conductance of the layer stack and the indices p and ap denote the parallel and antiparallel state of the ferromagnetic layers respectively.

The reason for the changing electrical resistance is the spin dependence of electron transport, which affects the scattering rates at film interfaces for spin-up (spin parallel to layer magnetization) and spin-down (spin antiparallel to layer magnetization) electrons. In case of film thicknesses smaller than the mean free path of the electrons, they move through all layers. For electrons passing the interface between the nfm layer and the fm layer with its magnetization antiparallel to the electron spin, the scatter rate is higher than for the electrons passing through the interface between the nfm layer and the fm layer with the magnetization parallel to the electron spin.

**Figure 5:**
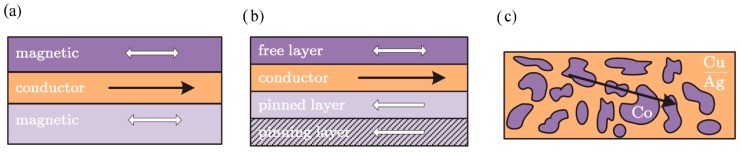
(**a**) Multilayer; (**b**) Spin Valve and (**c**) Granular System [3].

Although observed at first in multilayered thin films with interlayer exchange coupling (Figure 5a), any material combination with interfaces between a ferromagnetic and a nonmagnetic metal is theoretically able to display GMR. The relative orientation of the fm layers may result in IEC, but may also be achieved by the use of fm materials with different coercivities *H_c_* (soft and hard magnetic layers) or by pinning the magnetization direction of one of the fm layers using “natural” or synthetic antiferromagnetic layers (=GMR spin valve) (Figure 5b). GMR not only occurs in closed thin films. Another GMR structure is fm granular particles embedded into a nonmagnetic conductive matrix (granular GMR), which allows for spin dependent scattering of electrons at the particle–matrix interface (Figure 5c).

Simple multilayer systems show magnetoresistance 10%–80% [2,18] and switch their state at relatively high fields, as the external field has to overcome the interlayer exchange coupling. Spin valves are sensitive at low fields of around a few kA/m, as the free layer can rotate with the external field (mostly) unhindered and may achieve MR of up to 20% [19]. In granular systems, the behavior is strongly influenced by the production process and they have been reported to show under 10% up to 40% MR [2,20].

## 5. Designs and Commercial Applications of GMR Sensors 

**Figure 6:**
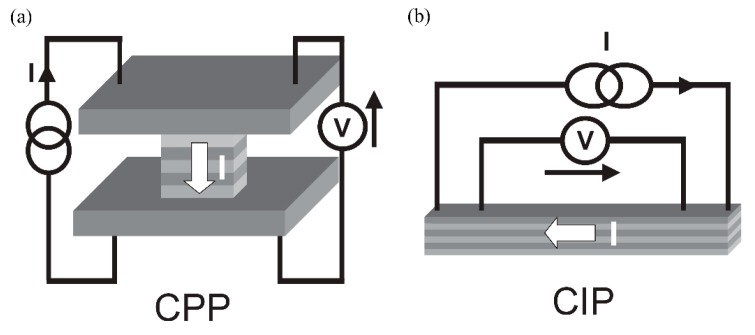
(**a**) Current-in-plane (CIP) and (**b**) current-perpendicular-to-plane (CPP) configurations in GMR layers [3].

The GMR effect can be measured with the electrical current flowing in the plane of the thin films or perpendicular to it, which is called current-in-plane (CIP) and current-perpendicular-to-plane (CPP), respectively (Figure 6). In most industrial applications, the CIP setup is used, as the film resistance in the CPP configuration is very small due to the very thin layers and therefore not easily to detect [21]. While the noise decreases in the lower-resistance CPP layers, so does the signal itself, resulting in low signal-to-noise ratios. Additionally, the fabrication process for CPP is more complex than for the CIP configuration, which means that fabrication time and costs increase.

In simple models, the conductance of the CIP configuration can be calculated as parallel circuit, while the CPP configuration is modeled with the layer resistivities in a series circuit.

As common for all resistive sensors, a design of meander shaped sensor elements connected in a Wheatstone bridge is used in most cases, in order to account for shape anisotropy and temperature compensation. Whether a half or a full bridge setup is used depends on the materials and layer structure as well as the application itself.

As for the production process, a wide range of technologies has been used to deposit the necessary layers, e.g., evaporation [22,23,24], dc and rf sputter deposition [25,26,27,28], ion beam sputtering [29,30,31] and molecular beam epitaxy (MBE) [32,33,34]. Important design parameters are among others the film thickness of each individual layer, the interface sharpness, crystallinity, grain size and other material properties describing the magnetic, thermal and mechanical behavior (e.g., coercivity, Curie temperature, Néel temperature, film stress, magnetostriction, *etc.*).

GMR sensors are employed for angle, speed and position sensing, e.g., in automotive applications as well as magnetic field and electrical current sensing in many industrial applications. In biological methods, GMR sensors implemented in Lab-on-a-Chip devices detect magnetic nanoparticles that are used as tags for different biomolecules [5,35,36]. The strongest impact of GMR technology was achieved in magnetic storage technology, as is well known. Special applications with an emphasis of three dimensional field measurements and high temperature applications will be outlined in Section 6.3 and Section 6.5, respectively. The work done at the IMPT in this context will be described in Section 6.4 and Section 6.6.

## 6. Current Developments

A substantial share in the scientific activity of the Institute of Micro Production Technology (IMPT), formerly Institute of Micro Technology (imt), has been dedicated to the development and the investigation of magnetoresistive sensor devices since its establishment in 1992. Consequently, various sensor devices have been studied, designed, fabricated and implemented throughout the last two decades. An innovative sensor device based on the AMR effect was published in 1999 [37]. This device consisted of two magnetoresistive permalloy elements connected in parallel. This circuit arrangement was chosen in order to obtain a Wheatstone half bridge, which generates linearized and temperature compensated sensor signals. Furthermore, the sensitive elements featured a high degree of shape anisotropy to ensure a steady state of magnetic domains, thus enabling a very high signal-to-noise ratio. First investigations on the manufacture of giant magnetoresistive thin films started in the late 90s [38] and led to the development of a process sequence for the realization of GMR spin-valve multilayers consisting of a Cr/CrMnPt/NiFe/Cu/NiFe thin film stack. An exemplary sensor device utilizing such a multilayer was presented in 2007 [39]. This device consisted of four single GMR elements arranged in a Wheatstone bridge and was used to determine the sensor characteristics of the developed GMR multilayers. 

Starting from these preliminary works, current research focuses on the advancement of MR sensors to meet the specific demands of industrial applications. In doing so, the scientific focus of the IMPT fits well with the zeitgeist of current research in the field of AMR sensors that favors the topic of system integration over investigations on the fundamental physics of the AMR effect itself. Starting from 1990, fundamental studies on MR effects have mostly been carried out concerning “newer” MR effects, such as tunneling (TMR) or colossal (CMR) magnetoresistance [40], since fundamental research on AMR has been performed for several decades [41] and the underlying physics are well understood by now. 

Advances regarding the system integration of AMR sensors cover fundamental applications like three-dimensional measurements of the earth magnetic field [42] and practical tasks like AMR sensor networks installed in traffic control or parking space detection outdoor [43]. An interesting example of AMR application in biomedicine is given by [44], who evaporated 90 nm thick permalloy layers on 100 nm thick SiO_2_ layers, which formed a rolled up structure and could be used to sort and monitor single cells, that have been magnetically labeled with Fe_3_O_4_ nanoparticles.

Another aspect of current research is to enable MR sensors for fields of application that could not yet be addressed due to design limitations of such sensors. Within this aspect, efforts are made to realize MR sensors on flexible substrates, permitting the mounting on curved surfaces as well as the realization of very thin, flexible devices. Likewise, the optimization of MR sensors in respect to functional capability under harsh environments is another issue on the path to find new opportunities for the application of MR sensors. 

### 6.1. MR Sensors on Flexible Substrates

The field of flexible electronics is a growing and promising market [45]. Since flexible electronics can be manufactured at low cost and feature high mechanical flexibility in use, they are interesting for a number of applications like flexible circuit boards [46] thin film solar cells [47], transistors [48] and more. 

When coating flexible polymers with metals, the polymer’s surface roughness will influence the microstructure of the metallic layer [49]. Furthermore, stress will be induced in metallic coatings on polymers due to two main reasons: strongly differing thermal expansion coefficients cause thermal stress and intrinsic stress appears because crystallographic flaws are built into the layer during deposition (applying for vacuum deposited layers) [50]. These impacts on the metallic layer will as well influence its magnetic performance [51]. The technical challenges caused through the interaction of metallic thin films with their substrates seem to be enormous, which is most properly one reason why AMR sensors on flexible polymers are hardly a topic of interest in current research activities.

Nevertheless, some publications in the field of GMR sensors on flexible polymers have been done. IBM, who first commercialized the GMR sensor in 1997 [52], released two papers concerning flexible GMR sensors with lower GMR effects compared to conventionally fabricated ones [53,54]. Siemens investigated in cooperation with the universities of Regensburg and Bielefeld strain gauge sensors based on the GMR effect that were manufactured on flexible polyimide. The main disadvantage arose from stress that appeared in the polyimide. The group around Schmidt from the University of Dresden is specialized on flexible GMR sensors. They enhanced the GMR performance by decreasing the polyimide’s surface roughness. Furthermore, they ball milled a sputtered GMR stack and mixed it with a polymeric binder. The paste is applied by brush painting and could generate a GMR effect when exposed to a magnetic field. This concept could lead to a printable GMR sensor when the powder is immersed in a solution. Most recently they presented a concept using transfer printing: a silicon carrier is coated with a sacrificial layer on which the GMR elements are deposited on and a pre-stretched strip of polydimethylsiloxane (PDMS) is then laminated onto this GMR layer. When the strip is released from the silicon, the GMR sensor is transferred to the PDMS showing a high stretch ability without cracks in the functional GMR layers. Additionally, a study using electroplated GMR layers on conductive polymer layers have been presented by Yan *et al.* [55]. 

### 6.2. Flexible AMR Sensors at the IMPT

#### 6.2.1. Flexible AMR Sensors for Industrial Applications

**Figure 7:**
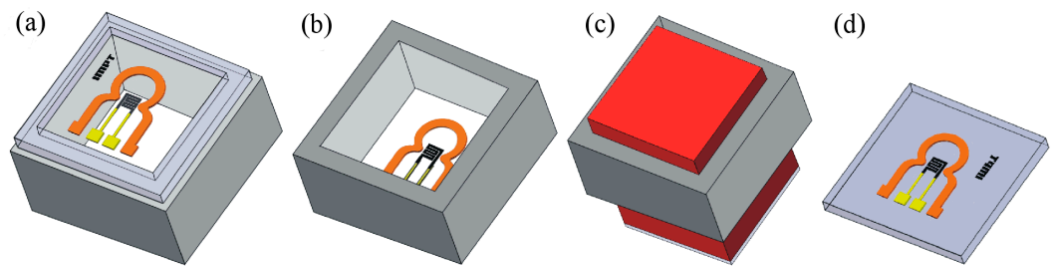
(**a**) The fabricated sensor is spanned using a silicon frame. By turning the frame upside down (**b**) and using a stamping tool (**c**), the sensor is separated (**d**) [58].

A manufacturing process that can be applied for the realization of flexible MR sensors has been developed at the IMPT. In this process, a photosensitive polyimide precursor is spin-coated on a 300 µm thick silicon wafer. Holes for contact pads are developed into the polyimide and a hard bake is carried out at 350 °C. The MR sensor layers are deposited on the hard-baked polyimide, and covered with another polyimide layer, which is hard-baked as well. Afterwards the backside of the silicon wafer is partly removed in a deep reactive ion etching process. This is the key element in the fabrication of the sensor. It creates very straight flanks in the silicon revealing a silicon grid of 200 µm width. The frame spans the manufactured sensors and enables an easy separation by using a stamping tool (Figure 7) [56,57,58].

In order to enable the process for a commercial fabrication of flexible AMR sensors, the deformations during the separation process of the sensor have been evaluated by measurements using a white light interferometer. Additionally, the mechanical strains have been simulated using ANSYS^©^.

**Figure 8:**
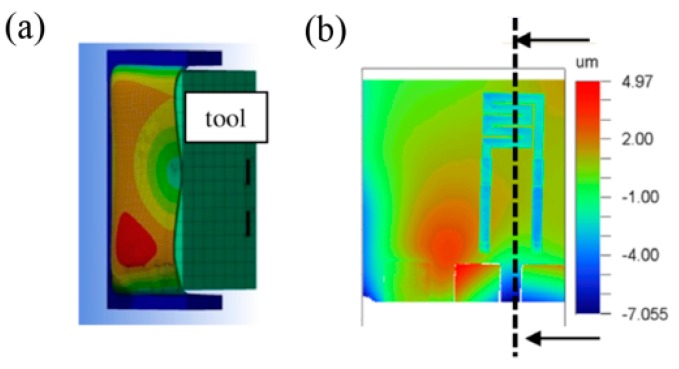
(**a**) Cross section of a simulated single sensor connected to the silicon grid (blue) when lifted by the tool (green); (**b**) Top view of the sensor’s deformation caused by a planar tool detected by the white light interferometer [59].

Figure 8 shows an angled view of the simulated cross section of the flexible sensor element on the silicon grid in comparison to a white light interferometer measurement; both during the separation process. The degree of deformation is demonstrated by a color grading from blue, indicating no deformation, to red, indicating a maximum deformation of about 12 µm.

Simulation and measurement equally showed a non-uniform deformation with a maximum deformation occurring in the lower left corner above the contact pad causing damages in the flexible substrate. Thus, in order to prevent damaging of the sensors during the separation process, a movable counterpart has been introduced to stabilize the flexible sensor during the separation.

**Figure 9:**
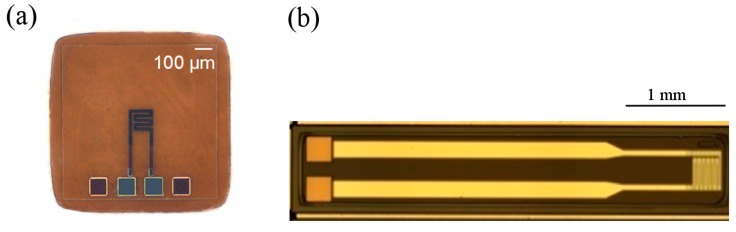
Evolution of a modular micro sensor (**a**) from a first generation to (**b**) second generation with larger feed cables, enlarged contact pads and a meander shaped sensing area with an increased number of turns [59].

After establishing the manufacturing process, the AMR sensor design has been modified in order to improve the magnetoresistive performance of the sensor (Figure 9) [60].

**Figure 10:**
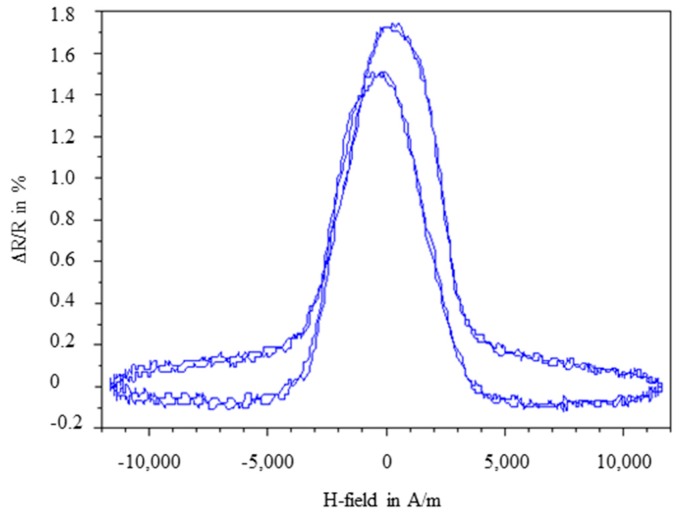
Ratio of electrical resistance change over an alternating magnetic field measured with a magnetic flux meter.

The manufactured flexible AMR sensors have been characterized in a magnetic flux meter showing an AMR effect of about 1.6%. In Figure 10 the evolution of the relative change of resistance of an exemplary specimen in an alternating magnetic field is shown. In an ideal case, the change of resistance should be identical for the alternation from negative to positive and the alternation from positive to negative. However, as shown in Figure 10, the curves are shifted and the maximum of magnetoresistivity is not reached when the magnetic field equals zero as predicted by theory. This unusual behavior is described as a distortion of anisotropy in literature [61] and can be explained by stress-induced anisotropy inside the functional layer of the AMR sensor, due to interactions with its polymer substrate. 

In conclusion, a reliable fabrication process for sensors on flexible substrates has been introduced serving as a basis for the industrial production of flexible AMR sensors. Such flexible AMR sensors offer outstanding opportunities: the introduced sensors feature a thickness of only 15 µm, making it possible to apply them in locations that are very difficult to access, e.g., extremely thin air gaps. In comparison, the thickness of commercial silicon substrates ranges from 150 to 300 µm. A reduction of weight goes along with the reduction of thickness of the substrate and can be of interest for portable consumer electronics. Further advantages of these sensors arise from their flexibility, making rough and uneven undergrounds possible places of installation. 

#### 6.2.2. AMR Sensor for Magnetic Storage Application on Technical Surfaces

Another example for the application of a flexible AMR sensor represents the magnetic storage of data on a technical surface. Inspired by classical hard drive magnetic storage technologies, a read/write head was developed in order to obtain a method to store critical product information intrinsically on a component. This will help a manufacturer as well as a user to identify and better apply a component [62].

By analogy with the classical hard drive magnetic storage technologies [63,64], a write head that generates a magnetic stray field near the air gap in order to magnetize the storage medium as well as a sensor functioning as a read head were developed and tested successfully [63]. 

Since the method was supposed to be applied in a conventional production environment with the occurrence of small vibrations, mechanical shocks and other disturbances, whereas a head-medium-spacing less than 25 µm was demanded in order to achieve sufficing data densities, a flexible read/write head was proposed as an improved solution. 

**Figure 11:**
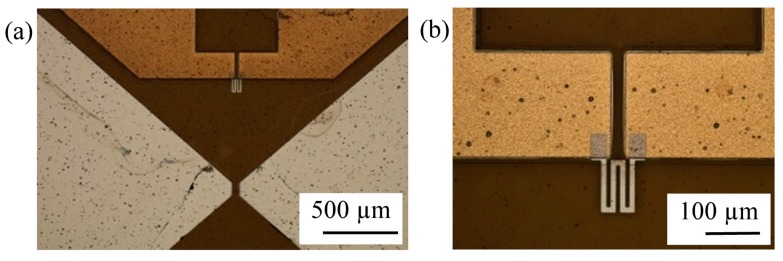
(**a**) Head poles of a read/write head; (**b**) MR element above head poles.

This flexible read/write head comprising all of its main elements, *i.e*., a soft magnetic head pole and a MR element, was fabricated on the same flexible substrate using solely thin film techniques. As flexible substrate, Kapton^®^ foil was used due to its outstanding physical properties [65]. Subsequent to preliminary works [66], multi-layer structures such as the soft magnetic head poles and the MR element have been fabricated on the Kapton^®^ polyimide film (Figure 11).

Generally, giant magnetoresistance (GMR) and tunnel magnetoresistance (TMR) elements as sensors for read heads are standard in magnetic data storage due to their operation range in high fields. However, their multi-layer structures are complex and their layer thickness must be strictly controlled. Benefits of those MR elements are promising in very high areal data density applications in hard disk drives with a very low head-medium spacing of the order of tens of nm. However, for the presented application, the head-medium spacing is in the order of tens of µm. Due to this spacing, data density is low and hence an anisotropic magnetoresistive (AMR) element can serve as a sensor as well. Thus, an AMR element was chosen as read element due to its simple fabrication. 

**Figure 12:**
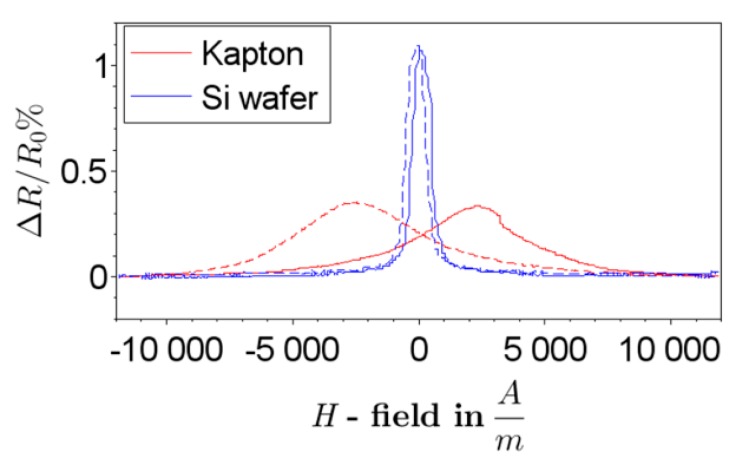
Ratio of electrical resistance change to basic resistance of AMR sensors on a Si-Wafer substrate (reference) and on a Kapton^®^ film; a solid line is obtained by sweeping a magnetic field *H* from the minimum to the maximum and vice versa for a dashed line.

Aforementioned stress transfer from flexible polymers into metallic layers is observed in this case as well and has been further investigated in [67]. It was found that stress induced in the substrate during the lamination of Kapton^®^ foil onto a carrier strongly influenced the direction of magnetization (*M*) of a NiFe layer and acts as bias force, as predicted by stress anisotropy. Characteristic curves of AMR sensors on a Si wafer substrate and on a Kapton^®^ film are shown in Figure 12. A magnetic field was applied in plane and perpendicular to an easy axis of the meander shaped structure. In case of the Kapton^®^ film, peak shifts were observed indicating a large deviation of *M* from the expected easy axis of magnetization. This deviates from the expected behavior of AMR sensors fabricated on Si-wafers. As a result, performance of a read head is affected, and hence careful treatments for readout signals are required. 

In summary, the main drawback for the realization of flexible AMR sensors is the impact of surface roughness and stress on the deposited metallic layer, influencing its magnetic properties. Nevertheless, flexible AMR sensors will offer new opportunities for the integration of this kind of sensors in industrial applications, providing an effective tool to gain more information in locations that are not accessible up to date. 

### 6.3. MR Sensors for Three-Dimensional Measurement

The capability to measure magnetic fields concerning their strength and direction is useful for a number of applications, especially when allowing for field measurement in three spatial directions integrated into one single miniaturized sensor chip in order to enable high alignment accuracy of the spatial axes and integration into portable devices and for other applications with space restrictions. This has been realized in different ways over the last decades, e.g., using sensor technology based on the Hall effect [68,69,70] or piezoresistive detection of mechanical stress induced by change of magnetization in a magnetic material [71]. 

In the last years, investigations were carried out with the goal of creating three-dimensional measurement capabilities using magnetoresistive sensors as they exhibit advantages like high sensitivity, small dimensions and low power consumption. 

The AMR effect was used to create a sensor capable of sensing a field component perpendicular to the substrate plane in the low-field range [72]. This was achieved by grooving the Si substrate with an anisotropic KOH etch and then depositing one AMR sensing element onto the substrate plane and two additional sensing elements into the V-shaped grooves, resulting in an inclination of these sensors in relation to the substrate plane. Thus, they are able to measure magnetic fields with a field component perpendicular to the substrate plane. This component was obtained by linearization of the inclined sensors using an external field along the plane and connecting them in a Wheatstone bridge. With this setup, a rejection rate of 100:1 for an in-plane field was achieved and a spatial resolution of 50 µm for a potential three-dimensional magnetometer was predicted.

Recently, the development of a three-dimensional GMR sensor utilizing a ferrite flux guide in order to redirect the field perpendicular to the in-plane direction into the sensing plane was published [73]. In the beginning stood the simulation and experimental validation of the response of a setup of two pairs of commercial dual bridge GMR sensors arranged on a cross shaped printed circuit board (PCB) with a cylindrical flux guide in the center. It was possible to switch between in-plane and out-of-plane measurement by modulating the phase of the modulation currents of the modulation coil wrapped around the sensor packages for each sensing direction from 0° to 180°. This prototype with a footprint of around 20 × 20 mm^2^ was developed further as a single bridge device in order to reduce the device dimensions as well as the power consumption and cost incurred by fabrication [74]. Four GMR spin valves were arranged around a cubic ferrite flux guide on a PCB and connected in a Wheatstone bridge. The out-of-plane flux component is bent at the edge of the flux guide, so that the spin valves can detect the created in-plane component. The working principle for a full bridge configuration is analytically presented as an example and implemented in the new prototype. With this setup, it is necessary to switch between the sensing directions. The authors investigated an alternative way by measuring the individual spin valve resistance changes and using these to calculate the bridge output. Using this device to measure a DC magnetic field resulted in high hysteresis and a shift of the individual spin valve outputs away from zero field. This was avoided by applying a modulation signal with an AC and a DC component. By adjusting the latter, the operation point could be optimized regarding linearity. In doing so, a linear magnetic field sensor device with three-dimensional measurement capabilities and low hysteresis could be implemented. As the in-plane sensitivities varied from each other due to alignment errors and the device dimensions are still rather big, the authors detailed future work comprising the transfer of the described sensor design onto a single chip using micro fabrication. Another similar device was investigated by this group, composed of three commercial GMR sensors mounted on a PCB around a cylindrical flux guide, wound with coils in order to provide a modulation signal [75]. For an arbitrarily applied external field with components in all three spatial directions, a voltage-to-field transfer matrix was devised and the matrix elements were determined by way of calibration. Verification of the azimuth response revealed that the transfer matrix allowed real-time calculation of the field components. The device dimensions were not given; further works were proposed in order to minimize the device features and to investigate the influence of non-ideal effects.

### 6.4. 3D GMR Sensor at the IMPT

Electric motors have become more and more important over the last several years, especially when considering the increasing relevance of e-mobility. The control of the magnetic flux density in the air gap between rotor and stator of an electric motor has a significant influence concerning the efficiency factor. If the magnetic flux density in the air gap is known, it is possible to improve the control and thus, increase the dynamic response and decrease energy consumption. The air gaps are usually very small, measuring a few hundred µm for systems of medium output power. This impedes the measurement of the magnetic flux, as the packages of commercial magnetic field sensors are usually much higher (around at least 1 mm). Within the scope of the joint project UltraMag, funded by the German Federal Ministry of Education and Research, we developed a magnetic field sensor with three-dimensional measuring capabilities based on packaged GMR with a height of 250 µm.

**Figure 13:**
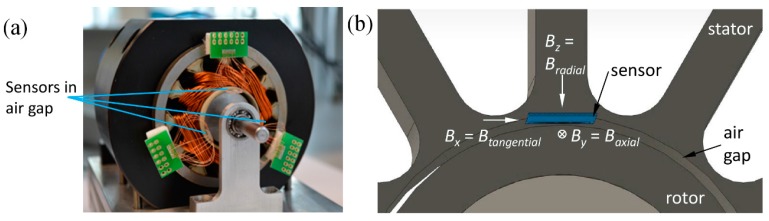
(**a**) Actual and (**b**) schematic depiction of a motor air gap with inserted magnetic field sensor.

In this case, the simultaneous measurement of the magnetic flux components in radial and axial direction (Figure 13) is of interest for use in an adapted control system.

The sensor described here is based on GMR spin valves due to their high sensitivity and linearity. Soft magnetic flux guides are used to attenuate and redirect the (external) magnetic flux in the air gap [76,77,78,79,80,81,82].

The simulation-assisted design of the soft magnetic flux guides was done in cooperation with our project partners from innomas GmbH. The magnetic flux has to be attenuated from the range of a few hundred mT to the measurement range of the spin valve sensors; the flux component perpendicular to the substrate plane has to be redirected into the chip plane, allowing the use of the exact same spin valve elements as for the flux components parallel to the chip plane.

**Figure 14:**
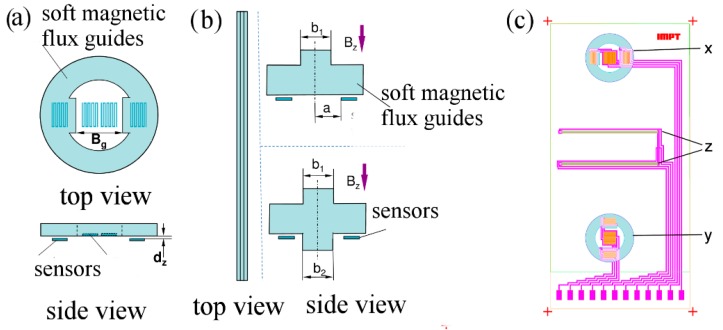
Geometric shapes of the flux guides (**a**,**b**) and sensor layout (**c**).

The design process yielded a ring-like shaped flux guide with short bars on the inner radius (Figure 14a) for measuring the in-plane flux in the *x*- and *y*-directions. The meander patterned spin valve elements are connected in a half bridge configuration with the active elements in the air gap between the inner bars and the passive elements placed underneath the flux guides in an area with close to zero flux. For the measurement direction perpendicular to the substrate plane (*z*-direction), we designed a rectilinear geometry due to fabrication limitations of curved patterns. These flux guides are patterned in a line and have a T- or cross-shaped profile (Figure 14b). The spin valves in this setup are connected in a full bridge and placed underneath the horizontal bar of the flux guide. The complete sensor layout is depicted in Figure 14c.

**Figure 15:**
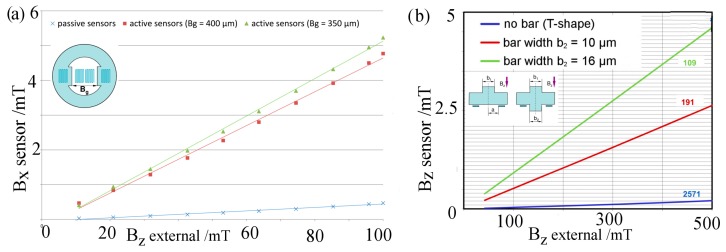
3D FEM modeling of attenuation factors for sensing direction parallel (**a**) and perpendicular (**b**) to substrate plane (source: innomas GmbH).

The three dimensional finite element method (FEM) modeling was carried out with varying dimensions for the given shapes. For the *x*- and *y*-direction, attenuation factors of around 20 for active elements and over 200 for passive elements were predicted (Figure 15a). Figure 15b shows that the external flux in the perpendicular direction is redirected, leading to an attenuated detectable flux component parallel to the substrate plane. Depending on the chosen geometry and its dimensions, attenuation factors of around 100 up to over 2500 were predicted.

The GMR spin valves used here consisted of a very simple layer stack, using Fe_50_Mn_50_ as the antiferromagnetic, Ni_81_Fe_19_ as the ferromagnetic and Cu as the non-magnetic spacer layer. For the fabrication of the soft magnetic flux guides, a CoFe alloy with a high magnetic moment was electroplated. As the quality of the flux guides was crucial for the functionality of the sensor, investigations concerning the optimization of the electroplating process for the CoFe alloy were carried out. The goals were to increase the saturation flux density, *B_s_*, and to decrease the coercivity, *H_c_*, and the film stress [77,78,79,82]. 

**Figure 16:**
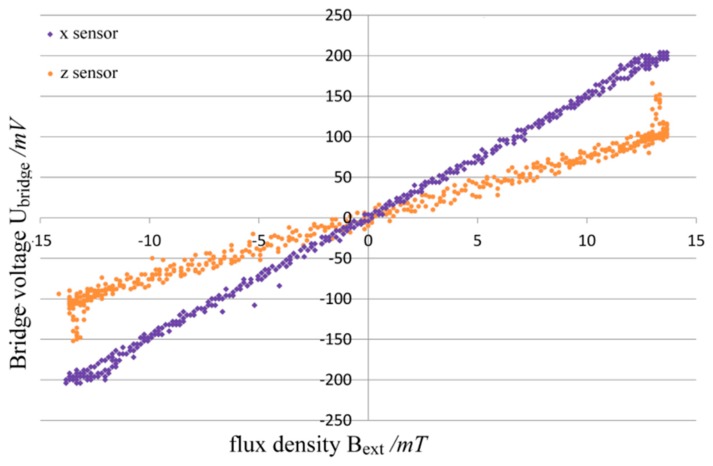
Transfer curves of amplified sensor output for *x*- and *z*-direction.

To evaluate the sensors, a homogenous field generated by Helmholtz coils has been utilized. The signal conditioning was done using a three-channel instrumentation amplifier. Exemplary transfer curves for measurements at a frequency of *f* = 50 Hz and a peak flux density of *B_pk_* = 14 mT are illustrated in Figure 16. The sensor signals are depicted for the *x*- and *z*-direction with the basic ring shape design and the cross shape, respectively. 

The non-linearity (0.16% in-plane and 0.23% out-of-plane) and hysteresis error (1.06% in-plane and 3.36% out-of-plane) as well as the sensitivity (0.3 mV/V/Oe in-plane and 0.18 mV/V/Oe out of plane) of the *x*-sensor are significantly lower than those of the *z*-sensor, which might be due to a higher non-linearity of attenuation caused by the geometry of the flux guide. This is an issue that necessitates further investigation. The reason for the spikes at the ends of the *z*-sensor transfer curve has still to be determined; investigations are still ongoing whether they are caused by the induction through the leads or by local saturation phenomena in the magnetic flux guides.

### 6.5. MR Sensors for High-Temperature Conditions

Magnetic field measurement at high temperatures and compatibility of MR sensors with high temperature fabrication processes poses a challenge in the context of magnetoresistive sensors. Thermal stability of a multilayer thin film sensor is limited on the one hand by diffusion and on the other hand loss of pinning, e.g., in the case of exchange bias. Diffusion creates mixed interfaces between the functional layers of the sensor, which is detrimental to the MR effect. The directional sensitivity of a MR device is often dependent on pinning of a ferromagnetic (fm) layer via exchange bias with an antiferromagnetic (afm) material. Above the material specific blocking temperature of the afm, magnetic order is lost and so is the pinning effect, resulting in a non-functional device. For the last 20 years, many investigations dealt with the realization of robust GMR sensors with reliable thermal stability in the range of over 200 °C, comprising of spin valve systems with IrMn, PtMn or NiMn as an afm due to their high blocking temperatures [83,84,85,86,87].

Most recently, the development of a monolithic GMR sensor for angle measurement in automotive applications was reported, for which long term stability in the temperature range of 175 °C were required [88]. The sensor under investigation, a spin valve with PtMn as the afm pinning layer, was designed for field measurement in the mT range a and exhibited no signal degradation during sensor lifetime (5000 h) for 175 °C, while for temperatures over 250 °C, the signal degraded significantly due to Mn migration from the afm layer towards the ferromagnetic layer. No reports for field measurement in the low field range (µT) under high temperature conditions were available.

### 6.6. High-Temperature GMR Sensor at the IMPT

For drilling activities at a depth of 4500 m and more, a navigation system is indispensable. However, the harsh environmental and drilling conditions are challenging for conventional electronics. Temperatures exceeding 150 °C, high vibrations, restricted space in the drill string and limited energy supply underground form the specification sheet. To navigate underground, different sensors are needed. Amongst others, magnetic field sensors are used. They work as a kind of compass by measuring the Earth’s magnetic field. Standard magnetic field sensors show deficiencies when applied at temperatures above 150 °C. At the IMPT, a magnetic field sensor has been developed which can operate at temperatures up to 250 °C. The sensor is characterized by a robust and small design and exhibits a relatively high resistance and low energy consumption.

The layer stack consists of materials that provide good thermal stability and high sensitivity. As ferromagnetic materials CoFe, convincing by its high Curie temperature, and NiFe, characterized by a low coercivity and anisotropy field, are used. Moreover, the pinned layer is designed as a synthetic antiferromagnet, which increases the thermal stability of the pinning and reduces the influence of the pinned layer’s magnetic field on the free layer’s sensitivity. As an antiferromagnet, NiMn, featuring a high blocking temperature, is chosen [89].

The layer stack was investigated with respect to its thermal stability (especially diffusion processes) in the unpatterned state. The investigations proved its thermal stability up to 250 °C for more than 660 h. Besides the thermal stability at 250 °C, the investigations revealed a correlation between the strength of the pinning field (the exchange bias field *H*_eb_) and the failure rate of the layer stack at higher temperatures [90]. It was observed that layer stacks with high exchange bias fields (*H*_eb_ ≥ 30 A/m) show better thermal stability. Investigations of the layer stack’s structure made evident that layer stacks with high exchange bias are composed of larger vertical grains, which nearly extend through the whole layer stack (from seed to the capping layer). Using the exchange bias field as an indicator for layer stacks featuring a high thermal stability, several parameter studies were conducted to determine the deposition parameters and layer thicknesses, which provoke a high exchange bias and presumably higher thermal stability. *H*_eb_-values of 80 kA/m could be realized [91].

In order to quantify the influence of structuring, the already optimized layer stack was etched into the meander shape and thermally stressed like mentioned above. Investigations on the meander level proved that after an initial slight degradation, thermal stability at 250 °C could be reached, for more than 500 h. Neither the basic resistance (*R_min_*) nor the maximum difference in resistance (*dR_max_*) alters in terms of measurement accuracy after this burn-in. Presumably the initial signal reduction is caused by initial diffusion processes at the sidewalls of the etched meander structures. 

**Figure 17:**
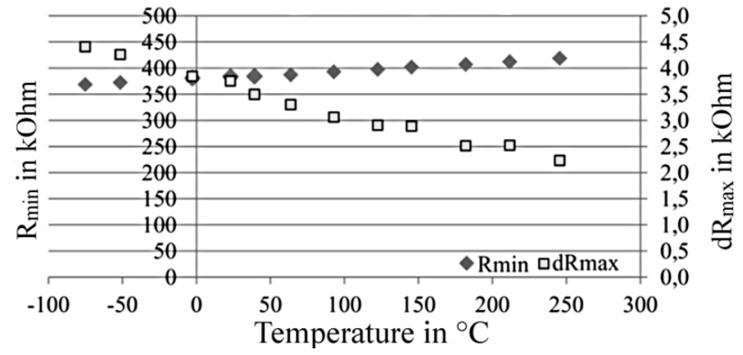
Temperature dependency of the meander structure’s signal [92].

Figure 17 shows the temperature dependency of the resistance at meander level within a temperature range from −75 °C to 245 °C. A nearly linear relationship can be observed. Whereas the basic resistance (*R*_min_) increases with temperature, the realizable resistance change (*dR*_max_) declines [92]. Relative to the signal at room temperature, the maximum resistance change is 117% at −75 °C and 60% at 245 °C of the room temperature signal. These reversible effect changes are the result of temperature dependent phonon and magnon scattering. By use of an adequate sensor design, this temperature dependency shall be reduced.

**Figure 18:**
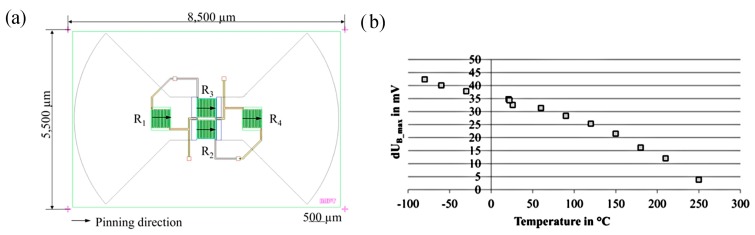
(**a**) Sensor design and (**b**) temperature dependency of the sensor signal.

The sensor contains flux concentrators in order to collect and amplify the Earth’s magnetic field. The concept of the sensor design is depicted in Figure 18a.

In order to prove the sensor’s long-term thermal stability, it was thermally stressed at 250 °C for 150 h. The room temperature change of bridge voltage was not affected, if the sensor was exposed to these temperatures for more than 140 h. Besides these long term tests, the robustness against thermal shock was tested according to MIL STD-883H D. The signal within the temperature range between −75 °C and 250 °C was investigated. The bridge voltage behavior is visualized in Figure 18b. 

The diagram reveals that the realizable voltage change decreases with temperature. This indicates that the shielding effect of the flux concentrators declines significantly within the investigated temperature range. In order to improve the shielding function, alternative materials for the flux concentrators, which were fabricated with a soft magnetic NiFe alloy for the time being, are under investigation. The focus of these investigations was on a ternary NiFeMo alloy.

It can be concluded that the developed magnetic field sensor can be applied at temperatures of up to 250 °C for several hours without irreversible degradation. However, further research work is needed to improve the signal’s dependency on temperature.

## 7. Conclusions and Outlook

The striking advantage of MR sensors, being highly sensitive over a broad frequency range at low cost, will ensure that the requirements of specific industrial applications will still be met efficiently with MR based solutions in the future. The aspect of low cost will lead to an enhanced use of polymers as substrates, which as well opens opportunities to manufacture printable electronic devices that can go without high-energy deposition technologies. Miniaturization of the devices will further be on topic to save space and to enhance functionality. The technology of magnetoresistive read heads could soon profit from developments of new MR concepts like Extraordinary MR, Coulomb Blockade MR and Tunneling Anisotropic MR.

The IMPT will continue its research in the field of MR sensors especially to offer solutions for specific industrial applications in future. These applications might necessitate sensors that deliver measurement data from difficult to access areas under harsh conditions (e.g., small air gaps in combination with high temperatures). Therefore, we see the demand for further investigations on thin magnetoresistive sensors on flexible polymers. 

Until the present, our work on flexible AMR sensors has led to promising results and will therefore be continued. The more complex GMR offer an interesting opportunity to further increase the field of application of MR sensors. Another promising concept being evaluated at the IMPT is to integrate an AMR sensor directly into a component by molding [93]. Direct depositing and structuring of sensors on technical surfaces is being investigated as well. The resulting advantages are the disuse of any substrate and adhesive layer. To realize the direct structuring of sensors on technical surfaces, a new sputter device has to be developed. Challenges during the development of such a device are to determine a method for structuring the layers [94] and the development of suitable insulation layers between the sensor and the rather rough, electrically conductive surface of the component [95].

MR sensor solutions for 3D measurements are an application of very high interest as well. The GMR sensor mentioned in Section 6.4 could be miniaturized to increase the precision of the device by placing the elements that measure the different axes as close as possible to each other on the chip. Hereby, the mutual interference of the single elements of the 3D sensors represents a risk of operational deterioration and needs to be prevented. 
